# Semiconductor-metal-semiconductor TiO_2_@Au/g-C_3_N_4_ interfacial heterojunction for high performance Z-scheme photocatalyst

**DOI:** 10.3389/fchem.2022.1050046

**Published:** 2022-10-21

**Authors:** Tingkai Hong, Shoaib Anwer, Ju Wu, Chonghai Deng, Hongmei Qian

**Affiliations:** ^1^ School Energy Materials and Chemical Engineering, Hefei University, Hefei, China; ^2^ Department of Mechanical Engineering, Khalifa University of Science and Technology, Abu Dhabi, United Arab Emirates; ^3^ Key Laboratory of Biomimetic Sensor and Detecting Technology of Anhui Province, West Anhui University, Lu’An, China; ^4^ School Energy Materials and Chemical Engineering, Hefei University, Hefei, China; ^5^ Department of Architecture and Civil Engineering, West Anhui University, Lu’An, China

**Keywords:** Z-scheme photocatalysts, Au/TiO2, G-C3N4, photocatalytic degradation, interfacial coupling

## Abstract

We designed an edge-sites 2D/0D/2D based TiO_2_@Au/g-C_3_N_4_ Z-scheme photocatalytic system consists of highly exposed (001) TNSs@Au edge-site heterojunction, and the Au/g-C_3_N_4_ interfacial heterojunction. The designed photocatalyst was prepared by a facile and controlled hydrothermal synthesis strategy *via in-situ* nanoclusters-to-nanoparticles deposition technique and programable calcination in N_2_ atmosphere to get edge-site well-crystalline interface, followed by chemically bonded thin overlay of g-C_3_N_4_. Photocatalytic performance of the prepared TNSs@Au/g-C_3_N_4_ catalyst was evaluated by the photocatalytic degradation of organic pollutants in water under visible light irradiation. The results obtained from structural and chemical characterization conclude that the inter-facet junction between highly exposed (001) and (101) TNSs surface, and TNSs@Au interfacial heterojunction formed by a direct contact between highly crystalline TNSs and Au, are the key factors to enhance the separation efficiency of photogenerated electrons/holes. On coupling with overlay of g-C_3_N_4_ 2D NSs synergistically offer tremendous reactive sites for the potential photocatalytic dye degradation in the Z-scheme photocatalyst. Particularly in the designed photocatalyst, Au nanoparticles accumulates and transfer the photo-stimulated electrons originated from anatase TNSs to g-C_3_N_4_ *via* semiconductor-metal heterojunction. Because of the large exposed reactive 2D surface, overlay g-C_3_N_4_ sheets not only trap photoelectrons, but also provide a potential platform for increased adsorption capacities for organic contaminants. This work establishes a foundation for the development of high-performance Z-scheme photocatalytic systems.

## Introduction

Ecological and energy issues are two of the world's biggest concerns today (Anwer et al., 2019; Li et al., 2021; Lu et al., 2022). Solar-assisted semiconductor-based photocatalysis is a potential and sustainable technology to address these concerns, as it utilizes free solar energy to generate clean energy and to remove organic pollutants from industrial effluent, which pose a major threat to environmental safety and human health (Krishna et al., 2022; Yao et al., 2022). The engineering of an artificial Z-scheme heterojunction is considered as an efficient method to improve redox capabilities because it provides an efficient photocatalytic performance through synergistic interactions between two different photocatalytic systems. Interestingly, low-dimensional 2D nanomaterials are generally recruited to obtain the large reactive surface area (Ong, 2017; Anwer et al., 2021). Regardless of the advances made in each of these areas, building a Z-scheme photocatalytic structure with suitable 2D materials’ geometry that combines these attributes into a single catalytic system remains a major challenge for the field experts (Das et al., 2022; Huo et al., 2022). Two key constraints to this endeavor are interfacial design and selective growth. It is necessary for two integrated semiconductors to have compatible band alignments, strong interface contacts, and a robust charge transfer route to create a Z-scheme heterojunction that is capable of strong redox reactions.

Because of their facet-dependent properties, extensive studies have been conducted on the preparation of TiO_2_ nanosheets (TNSs)-based SCs with large exposed (001) high-energy facets since Lu and his co-workers pioneered the preparation of a uniform anatase single crystal sheet with 47% (001) facets (Yang et al., 2008; Li et al., 2022; Wang et al., 2022). Theoretical calculations and experimental studies proved that the (101) facets are more reductive than the (001) facets because they can operate as potential reservoirs for photogenerated electrons, whereas the (001) facets, as oxidation sites, play the important role in photo-oxidation reactions (Pan et al., 2011; Zhang et al., 2018). It is realized that the synergistic effect of different facets could be an interesting fact that has been ignored in most research studies in the past. Thus, the surface heterojunction may provide a novel technique for building highly effective photocatalysts, which merits further research. For instance, noble metals (Pt, Au, Ag, Cu) that induce plasmons can act as excellent electron mediators when deposited on the surface of semiconductor heterostructures, promoting charge carrier transfer due to its localized surface plasmon resonance (LSPR) (Lu et al., 2019; Wang and Kowalska, 2022). The metal-free graphitic carbon nitride (g-C_3_N_4_) proved to be stable, non-toxic, and easy to prepare 2D material, that can effectively activate molecular oxygen and generate superoxide free radicals for photocatalytic degradation of organic pollutants (Zhao et al., 2018; Yang et al., 2022). For wide band semiconductors, g-C_3_N_4_’s narrow band gap is effective at absorbing visible light and it is relatively negative CB position ensures heterojunction charge transfer under visible light (Sakuna et al., 2022). Yang et al. took the benefit of combining the morphology control and defect engineering, and fabricated a Van der Waals heterojunction from ultrathin WO_3_•H_2_O and GCN NSs to achieve an efficient Z-scheme water splitting without sacrificing agents and cocatalysts (Yang et al., 2018). These methods are successful to build various types of Z-scheme photocatalytic systems. However, their primary limitation is the lack of a high-performance photocatalyst with a large reactive surface area and good charge separation. In particular, when depositing Z-scheme structures randomly or site-selectively, uncontrolled deposition of secondary semiconductor nanostructures usually insulates the surface of the first semiconductor, resulting an increase in reduction surface area at the expense of oxidation surface area or *vice versa*. The extrinsic recombination pathways will reduce the total charge separation efficiency even if the heterojunction itself is capable of charge separation. As a result, sacrificial agents are routinely used to assure photocatalytic function in such photocatalysts.

Herein, an effective charge separation, redox capability, and enlarge active surface area are achieved simultaneously by coupling 2D/0D/2D material-based TNSs@Au/g-C_3_N_4_ ternary Z-scheme photocatalysts. Precisely, crystalline TNSs with (001) exposed facets and 2D g-C_3_N_4_ sheets were recruited as two-component materials. Using a diffusion and spatial confinement technique, Au NCs are initially decorated on (101) edge-facets surface of 2D TNSs. Then 2D g-C_3_N_4_ sheets was selectively chemically bonded on Au NCs and TiO_2_ edge sites by liquid phase chemical synthesis strategy followed by programmable annealing in N_2_ atmosphere. A second advantage of this unique design is the physical isolation of the redox reactive surfaces, allowing oxidation reactions to take place on the bare (001) facets of TNSs and reduction reactions to take place on 2D g-C_3_N_4_ sheets. In addition to acting as a mediator between two semiconductors, Au NCs bridged between them cause hot electron injection. Thus, ensure efficient Z-scheme charge transfer and speed up the relocation of photoinduced electron holes at the heterointerface, improving the photocatalytic performance. RhB and MO was used as an organic pollutant to explore the photocatalytic degradation performance of the prepared photocatalyst. Particularly, in the prepared photocatalyst a ternary TNSs-Au-g-C_3_N_4_ nanojunction based Z-scheme route was developed which boost the charge separation efficiency, and enhance the activated localized photoelectrons’ surface density. In short, the exposed (001) and (101) surface/inter-facet junction and the Au/TNSs interfacial heterojunction enables the active charge separation, coupled with 2D overlay of g-C_3_N_4_ sheets synergistically offer tremendous reactive sites for the potential photocatalytic dye degradation in the Z-scheme photocatalyst, this is far superior compared to single or binary photocatalysts. This work might offer a particular perspective on the construction of superior Z-scheme photocatalysts based on 2D/0D/2D ternary heterostructures for promising applications in environmental remediation and energy harvesting.

## Materials and methods

### Chemicals and materials used

Research grade tetrabutyl titanate (C_16_H_36_O_4_Ti, >97%), hydrofluoric acid (HF, 48%), ammonium tetrathiomolybdate (H_8_N_2_MoS_4_, 99.97%), Rhodamine B dye (RhB) (C_28_H_31_ClN_2_O_3_), and methylene Orange (C_14_H_14_N_3_NaO_3_S) were received from Sigma-Aldrich (St Louis, MO, United States). Urea (H_2_NCONH_2_, 99.97%), ethanol (C_2_H_5_OH, 99.8%), and Hydrogen tetrachloroaurate (III) (HAuCl_4_.4H_2_O, 99.99%) are obtained through Merck Millipore. Deprived of further treatment, all of the chemicals were utilized as obtained. A Milli-Q system was utilized to purify the water used in the studies.

### Preparation of crystalline anatase TNSs with large exposed (001) facets

Typical synthesis procedure involved adding 7.5 ml of tetrabutyltitanate and 0.9 ml of HF stock solution to a dried Teflon autoclave vessel at room temperature, followed by gentle stirring. The resultant solution is then put in an autoclave, heated at 190 °C for 18 h. Centrifugation was used to recover a yellow milky precipitate after the reaction had concluded, after that, it was thoroughly cleaned with distilled water and dried in an oven at 80 °C throughout the night.

### Preparation of edge-selective TNSs@Au heterojunction nanocomposite

An aqueous phase synthesis strategy with urea as a basifying agent was used to prepared TNSs/Au nanocomposite. Precisely, 150 mg of as-prepared highly crystalline anatase TNSs was thoroughly dispersed in 15 ml of ultrapure water under successive process of 40 min of ultrasonication followed by 60 min of vigorous stirring. After that, 4.2 mM HAuCl_4_.4H_2_O is injected to the milky solution, resulted in the formation of Au nanoclusters on the TNSs at random positions. In a next step to reduce the Au ions, urea is added in an amount of hundred times to the concentration of the Au precursor (Anwer et al., 2018). The resulting dark-yellow heterogeneous mixture underwent 3.5 h of thermostatically controlled heating at 80°C while being vigorously stirred. After the reaction was finished, the precipitates were separated and treated with ethanol and deionized water to wash. The collected sample is vacuum dried all night at 90°C in a kiln to produce randomly-deposited TNSs@Au with a 4 wt% Au loading. To get the edge-site selectively deposited TNSs@Au, the above-prepared randomly-deposited TNSs@Au was calcined from room temperature to 300°C in the N_2_ environment for 3 h at a flow rate of (30 ml min^−1^) and a heating rate of 3°C min^−1^. Finally, a light purple TNSs@Au heterojunction nanocomposite with selective edge-site deposition was obtained.

### Synthesis of graphitic carbon nitride (g-C_3_N_4_) sheets

Graphitic carbon nitride (g-C_3_N_4_) sheets were synthesized according to previous work. Briefly, a calculated amount of urea was added in a quartz crucible with a lid that is kept at 550°C for 4 h at a heating rate of 5°C·min^−1^ in an air-sealed tubular furnace under N_2_ atmosphere. Once the reaction was completed, the furnace is cooled down naturally and the obtained dark-yellow product was washed with ethanol and DI water several times. Finally vacuum-dehydrated at 80°C overnight and stored as a bulk g-C_3_N_4_ for further use.

### Fabrication of ternary composites TNSs@Au/g-C_3_N_4_

As solution A, 100 mg of freshly manufactured TNSs@Au powder was thoroughly dissolved in 10 ml of DI water. On the other hand, a calculated amount of g-C_3_N_4_ (2, 5, and 10 wt% of TNSs@Au) was dispersed in 20 ml ethanol with ultrasonication of 2 h as solution B. Then the solution B was poured into the solution A drop-wise under magnetic stirring. The mixed suspension was magnetic stirred for ∼20 h to complete the chemical encapsulate process. After reaction, the DI water was used to centrifuged and washed the vague-black color semi-gel precipitates suspension several times, and then overnight dried in a vacuum oven at 90°C. The final step was to anneal the resultant product in N_2_ for 3 h at 400°C in order to produce the desired TNSs@Au/g-C_3_N_4_ ternary nanocomposite.

### Morphological and structural characterization

A HITACHI H-7650 electron microscope (accelerating voltage of 80 kV) was used to obtain low-resolution TEM images of the prepared samples. To prepare a TEM sample, aqueous solution of the prepared materials was dropped onto a copper mesh (300 mesh, carbon support film) and dried at room temperature. FEI Tecnai G2 F20 S-Twin microscope operating at 200 kV and equipped with an X-ray energy-dispersive spectroscopy detector (XEDS) was used to perform HRTEM, element mapping, and EDS. X-ray photoelectron spectroscopy (XPS) examination was performed using a Perkin-Elmer PHI 5300 ESCA system (Mg K) at 250 W under a vacuum greater than 10^–6^ Pa to examine the main composition and chemical state of the produced samples. All XPS spectra's binding energies are standardized by the C 1s peak's 284.6 eV value. The crystallinity and phase purity of the prepared samples are investigated by powder X-ray diffraction (XRD) on a Bruker D8 Advance XRD, the diffraction patterns are measured using Cu K radiation (*λ* = 1.54178 Å). A Hitachi FESEM-4800 field emission scanning electron microscope (FE-SEM)) was hired to examine the morphologies and microstructures of the fabricated catalysts at a large scale. Shimadzu UV3600 UV-vis-NIR spectrophotometer is hired to record the UV-vis absorption spectra of the samples. The samples’ infrared spectrum was collected using an FTIR spectrometer (Bruker Tensor 27, Germany) with samples made from KBr pellets and spectra ranging from 4000 to 450 cm^−1^ acquired.

### Photocatalytic activity

The degradation studies of RhB and MO in an aqueous solution under visible light irradiation was carried out to assess the photocatalytic performance of the produced samples. A Xe (150 W) lamp was used with suitable filters to produce visible light (*λ*≧420 nm). The reactor and the light source were separated by distance of 10 cm. An amount of 50 mg of the synthesized catalyst was homogeneously dissolved in 100 ml of water to get 10 mg/L aqueous solution of RhB or in 50 ml of a 5 mg/L aqueous solution of MO. This solution was magnetically agitated in the dark for 30 min to ensure the creation of an adsorption/desorption equilibrium. Then, to illuminate the suspension, a Xe light with a UV cutoff filter was used. A 3 ml aliquots of the solution are collected, centrifuged, and filtered to get rid of sediments after predetermined time intervals. We measured the absorption spectra of RhB and MO aqueous solutions vs. irradiation time. The dye concentration in the solution was determined by its absorption peak intensity (for RhB at 554 nm and for MO at 464 nm). The UV-vis spectrum was assessed, and the absorbance of *λ*_max_ was used to calculate the degradation efficiency (%) as:

$$\text{Efficiency}\left(\%\right)=\frac{C_{0}-C}{C_{0}}\times 100\%$$

### Photocurrent response measurements

Measurements of the photocurrent were made using a typical three electrode quartz cell on an electrochemical workstation (CHI-660E, Shanghai, China), equipped with personal computer. As-prepared samples were deposited as aqueous slurries on ITO glass substrates to create the working electrodes (coating area was 0.25 cm^2^), then overnight air dried at 90°C. To test the photocurrent response in a 0.2 M Na_2_SO_4_ electrolyte solution under visible light on/off condition, a platinum wire is used as the counter electrode and a saturated Ag/AgCl served as a reference electrode. The anodic and cathodic photocurrents are analyzed under irradiation of a Xe light (150 W) fitted with a cutoff filter (AM1.5G, *λ* > 420 nm, and 100 mW cm^−2^) at a volatge of +0.8 V and -0.8 V, correspondingly. The power of incident simulative sunlight (AM 1.5G) was calculated by Spectroradiometer (LPE; Beijing Wuke Photoelectric Technology Co. Ltd. China). The reaction cell irradiated by the incident light was 0.034 m^2^. The intensity of the light over the irradiation surface was 79 mW cm^−2^. In the supporting information, Supplementary Figure S4 shows the spectrum obtained from the datasheet of the Xenon light source (150 W) used in this study.

## Results and discussion

**FIGURE 1:**
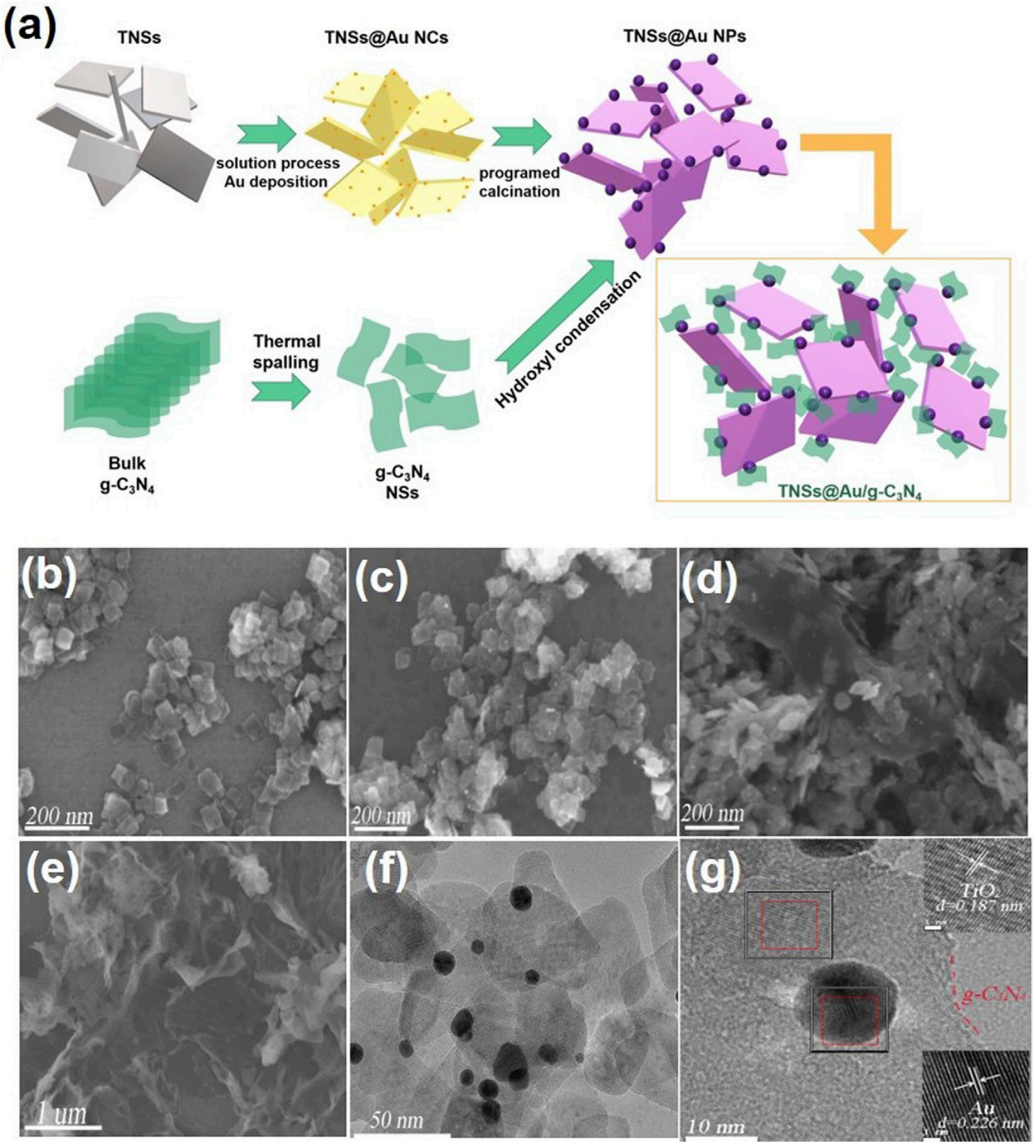
**(A)** Schematic illustration of the synthetic route for the preparation of TNSs@Au/g-C_3_N_4._ The SEM images of **(B)** TNSs, **(C)** TNSs@Au, **(D)** TNSs@Au/g-C_3_N_4_, **(E)** g-C_3_N_4_; and TEM **(F)** and HRTEM **(G)** images of TNSs@Au/g-C_3_N_4_.

**FIGURE 2:**
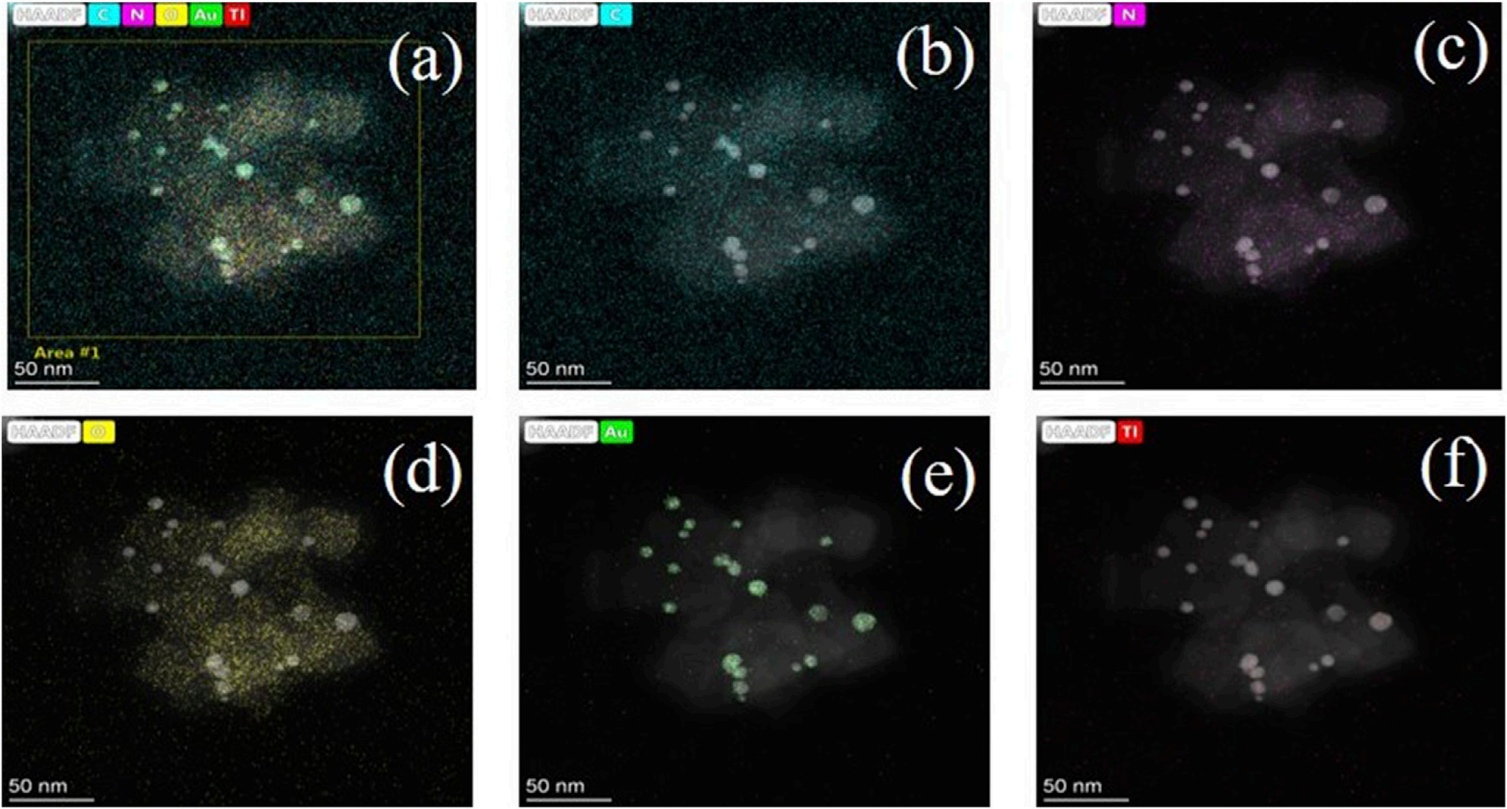
Corresponding elemental mapping: **(A)** Survey map, **(B)** C, **(C)** N, **(D)** O, **(E)** Au, and **(F)** Ti.

The synthesis process of TNSs@Au/g-C_3_N_4_ ternary nanocomposite is depicted schematically in Figure 1A. In the first step, we used the hydrothermal technique to create rectangular crystalline TNSs. After adding Au precursor to the milky white TNSs aqueous suspension, urea was added as a basifying agent to deposit Au nanocrystals *via* liquid-phase deposition method. The Au anions adsorbed on the surface of TNSs transformed into a pale-yellow Au nanocluster and dispersed randomly across the surface. Next, we were able to preferentially deposit Au NCs on the (001) exposed edge-site facets of TNSs as a result of *in-situ* programable calcination. Ostwald ripening of and nucleation growth resulted in the dark purple hue precipitates of TNSs@Au. Thermal spalling is used to create graphitic carbon nitride (g-C_3_N_4_) sheets in accordance with earlier research. To produce the desired TNSs@Au/g-C_3_N_4_ ternary nanocomposite, a particular quantity of newly made TNSs@Au powder was fully dissolved in DI water and drop-wise mixed with discrete quantities of g-C_3_N_4_ ethanol suspension while being magnetically agitated. For further information on the precise synthesis procedure, please refer to the experimental section. The SEM image of pure TNSs in Figure 1B exhibited a rectangular morphology with lateral dimension in range of ∼60–70 nm, and were ∼7 nm thin. Due to ultrathin 2D morphology these TNSs possess higher surface area compared to TiO_2_ NPs. SEM image in Figure 1C showed the distribution of Au NPs deposited on TNSs without affecting the morphology of primary TNSs. Interestingly, no significant change in the morphology of TNSs@Au were observed after introducing g-C_3_N_4_ NSs, see SEM Figure 1D that confirmed the successful synthesis protocol. While Figure 1E showed the SEM image of as-prepared g-C_3_N_4_ NSs. To cross-verified the morphology of the prepared composite and synthesis strategy, we performed TEM and HRTEM imaging of the TNSs@Au/g-C_3_N_4_ catalyst, the results were shown in Figures 1F,G respectively. It was observed that the TNSs were almost ∼60 nm, which is consistent with the SEM data. Moreover, dispersed Au NPs were clearly visible on TNSs. The calculated lattice spacing parallel to the transverse lattice was 0.187 nm due to the (001) facet of the TNSs (Chen et al., 2010; Shoaib et al., 2016). The high exposure (001) active surface on the TiO_2_ surface has the potential of promoting photoinduced electron-hole pairs. The lattice spacing of the Au NPs was 0.226 nm, which corresponds to the exposed (111) crystal facet. While the amorphous region was recognized as g-C_3_N_4_ structure, the crystal diffusion region at the phase-interface of Au, TNSs, and g-C_3_N_4_ was observed in the HRTEM Figure 1F, indicating that there is a strong interfacial contact between each component of the composite photocatalyst. The TNSs@Au/g-C_3_N_4_ catalyst's elements were mapped using HAADF-STEM in Figure 2. The elements in the mapping images for C, N, O, Au and Ti portrayed in different colors, proved the successful synthesis of TNSs@Au/g-C_3_N_4_ (Wang et al., 2020).

The XRD patterns in Supplementary Figure S1 showed that prepared TNSs, TNSs@Au, and TNSs@Au/g-C_3_N_4_ catalysts were composed of pure anatase phase of TiO_2_, as they are in perfect match with the reference patterns (PDF# 21–1272). In all prepared TNSs based catalysts samples, the crystallographic planes assigned to the observed diffraction peaks at 25.1°, 38.2°, 48°, 53.8°, 55°, 62.6°, 68.6°, 70.2°, and 75°are (101), (004), (200), (105), (211), (204), (116), (220), and (215), attributed to TiO_2_ anatase phase, respectively. (Naldoni et al., 2013). The diffraction peaks for Au composition, which were detected at 38.20°, 44.4°, 64.6°, and 77.5° in TNSs@Au and TNSs@Au/g-C_3_N_4_ samples were indicative of a face-centered cubic Au (PDF# 04–0784) (Bian et al., 2014). After deposition of Au NCs, the anatase phase is sustained without experiencing a phase transition, according to the XRD patterns peak indexing. Except for the distinct Au peaks in TNSs@Au, that confirmed an excellent crystallization of Au following heat treatment, no discernible difference between the XRD patterns of TNSs was found. Only a strong-shoulder diffraction peak at 13.4° and a weak peak at 27.4° were clearly visible for pure g-C_3_N_4_ catalyst (Chai et al., 2012). The weak diffraction peak at 27.4° indicates that the accumulation of conjugated aromatic rings was weak, that also proved the ultrathin thickness of g-C_3_N_4_ NSs. The characteristic peak at 13.4° corresponds to the diffraction of the (100) crystal surface diffraction of the g-C_3_N_4_, assigned to the orderly arrangement of the condensed arrangement (Sun et al., 2015). When it comes to TNSs@Au/g-C_3_N_4_ photocatalysts, the typical diffraction peaks were consistent with those of pure TNSs@Au NC and the supported g- C_3_N_4_ diffraction peak was not seen. This demonstrated that the supported g-C_3_N_4_ NSs were thin and homogeneous in thickness and that they had no effect on the crystalline structure of the TNSs@Au NCs. No other crystal phase peaks were detected in the XRD patterns, which implied that the high purity formation of the as-obtained photocatalysts. X-ray photoelectron spectroscopy (XPS) investigations were carried out to find out metal-support interaction and surface chemistry of the produced TNSs@Au catalysts. The Ti 2p, O 1s, Au 4f, and C 1s peaks are seen in XPS full survey spectrum of TNSs@Au (Supplementary Figure S2A), which corresponds to their respective binding energies. Two distinct peaks for Ti 2p_3/2_ and Ti 2p_1/2_ are visible in Ti 2p XPS spectrum (Supplementary Figure S2B) at 458.7 eV and 464.4 eV, correspondingly (Shoaib et al., 2017). Three prominent peaks with centers at 529.9 eV, 531 eV, and 532.1 eV, which correspond to the lattice oxygen (O_L_) of TiO_2_, the oxygen-deficient area O_v_, and chemisorbed oxygen species (O_c_), respectively, can be deconvoluted from the high-resolution O 1 s spectrum (Supplementary Figure S2C) (Naldoni et al., 2013). The high resolution XPS spectrum of Au 4f is shown in Supplementary Figure S2D, where the two main peaks at 87.0 eV and 83.3 eV were assigned to the Au 4f_5/2_ and Au 4f_7/2_ (Hu et al., 2022). Because cationic gold species have binding energies greater than 84.0 eV, the standard for metallic gold, the major Au 4f7/2 peak was found at 83.3 eV, which corresponds to metallic Au_0_ (Sun et al., 2020). The fact that the value was less than 84.0 eV supports the electrical interaction between Au NPs and TNSs by indicating that negative charge was transferred from TiO_2_ to Au NPs. (Wang et al., 2021). These XPS data provided more evidence that TNSs@Au was made up of Au and TNSs.

**FIGURE 3:**
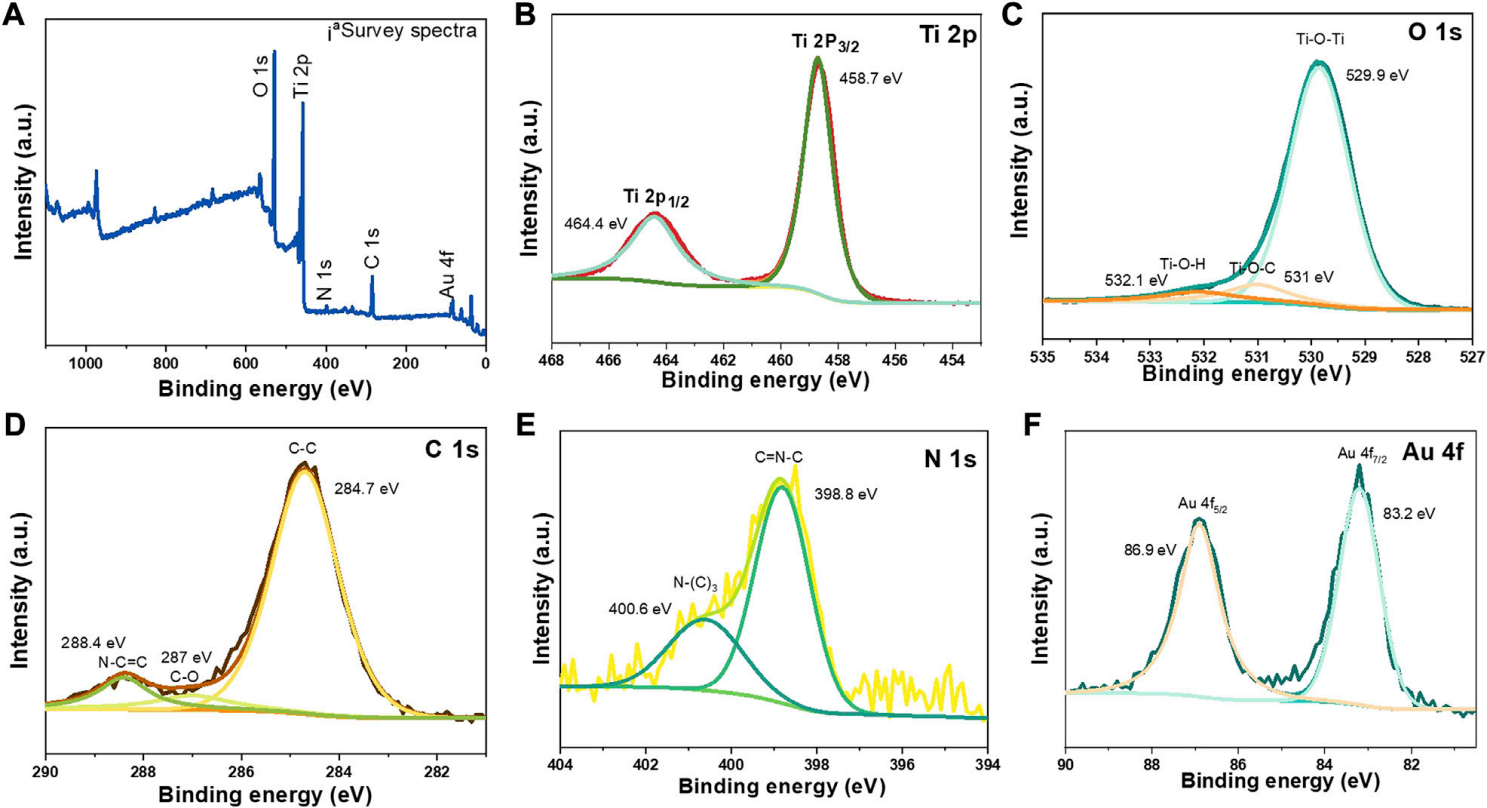
High-resolution XPS spectra of synthesized TNSs@Au/g-C_3_N_4_: **(A)** full scan survey, **(B)** Ti2p, **(C)** O 1s, **(D)** C 1s, **(E)** N 1s, **(F)** Au 4f.

The XPS examination was performed to look into the chemical make-up and valence states of the components added to TNSs@Au/g- C_3_N_4_ photocatalysts (see Figure 3). As shown in Figure 3A, the intensity of N1s and C1s XPS peaks have an obvious increased compared to Supplementary Figure S2A, ascribed to the introducing g-C_3_N_4_ NSs. As for the position and intensity of Ti 2 p (Figure 3B) and O 1s (Figure 3C) characteristic peaks are concerned, no obvious change in peaks was observed. The C1s XPS spectra of TNSs@Au/g-C_3_N_4_ (Figure 3D) are subjected by two distinct peaks of elemental carbon at 284.6 eV accredited to sp^2^ C‒C bonds and t 288.4 eV peak corresponded to sp^2^-bonded carbon (N‒C=N) of carbon nitride in graphitic phase, individually (Marchal et al., 2018; Niu et al., 2018). An oblique peak was found at 287.0 eV in the TNSs@Au/g-C_3_N_4_ catalyst, attributed to O‒Ti‒O‒C‒N bonds formed at g-C_3_N_4_-TNSs@Au interface (Zhang et al., 2018). Figure 3E shows the decomposition of the N 1s XPS spectrum of the TNSs@Au/g-C_3_N_4_ catalysts into two main peaks, with one peak assigned to sp^2^-hybridized nitrogen of the C=N‒C functional group at 398.8 eV and the second peak allocated to N‒(C)_3_ ternary nitrogen at 400.6 eV (Tay et al., 2016; Wei et al., 2018; Wang et al., 2021). In the case of TNSs@Au/g-C_3_N_4_, the core-level XPS spectra of Au 4f (Figure 3F) was discriminated into double distinct peaks that correspond to Au 4f_7/2_ and Au 4f_5/2_, are focused at 83.2 and 86.9 eV separately (Tada et al., 2006). A slight negative drift in the binding energy of Au 4f_7/2_ compared to 83.3 eV of the Au-TNSs in the TNSs@Au/g- C_3_N_4_ catalyst, is ascribed to chemical interaction of Au NPs with TNSs and g-C_3_N_4_ NSs.

**FIGURE 4:**
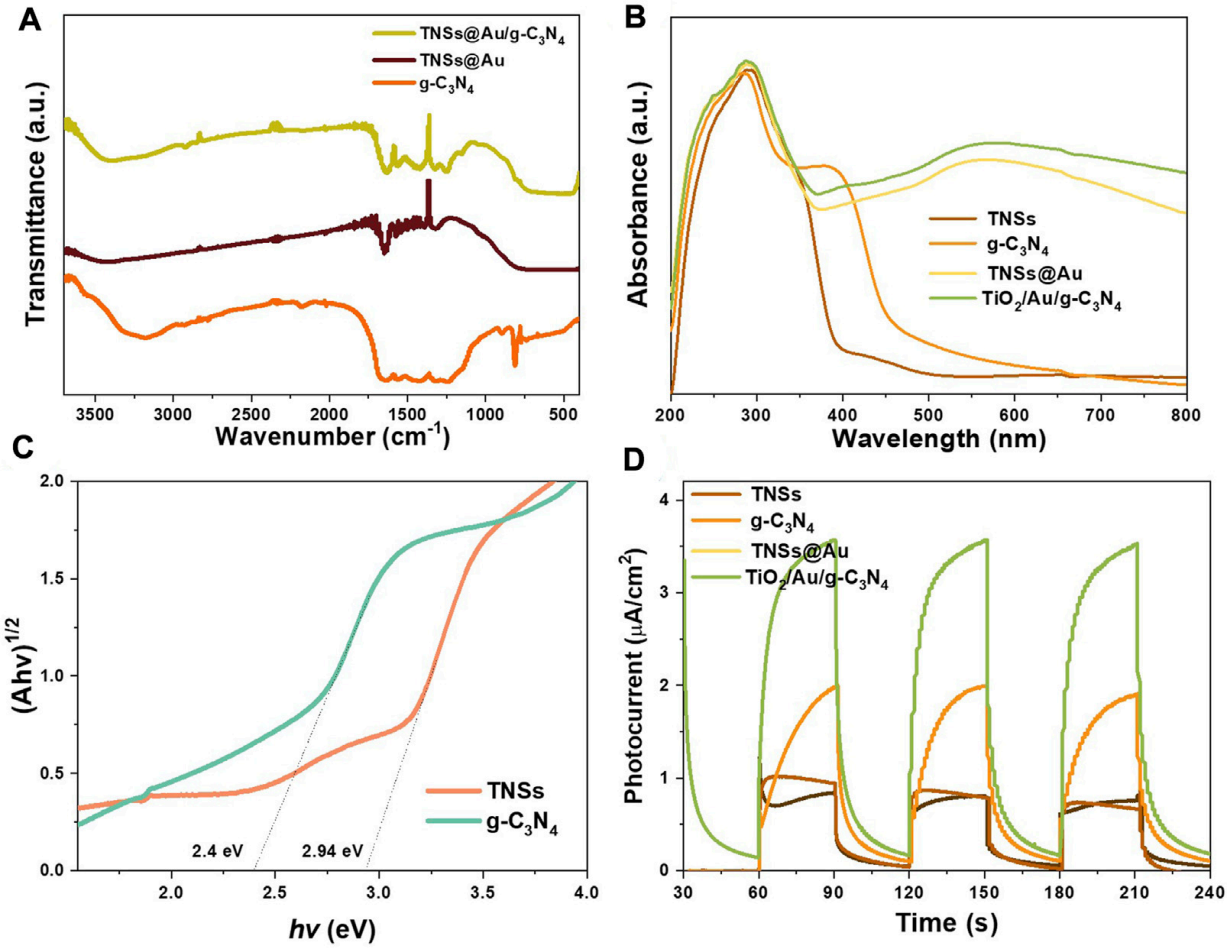
**(A)** FTIR spectra of as-prepared g-C_3_N_4_, TNSs@Au, and TNSs@Au/g-C_3_N_4_ photocatalysts. **(B)** UV–Vis DRS spectra of TNSs, g-C_3_N_4_, TNSs@Au, and TNSs@Au/g-C_3_N_4_ catalysts. **(C)** Kubelka-Munk transformed reflectance spectra. **(D)** Transient photocurrent responses of the prepared photocatalysts samples.

The surface chemical bonds and functional groups contained in the produced catalysts are investigated using Fourier transform infrared (FTIR) analysis (see Figure 4A). There was a collection of distinctive absorption bands in the range of 1200–1700 cm^−1^ for pure g-C_3_N_4_ NSs. The wide bands from 3048 to 3310 cm^−1^ were originated by the terminal N‒H or NH_2_ group stretching vibrations of g-C_3_N_4_. The absorption bands at 1249 cm^−1^, 1330 cm^−1^, 1411 cm^−1^, 1456 cm^−1^, 1572 cm^−1^, and 1634 cm^−1^ are attributed to the stretching vibration modes of heterocyclic C‒N (Md Rosli et al., 2018; Zhou and Qiu, 2019). The characteristic peaks located at 807 cm^−1^ represents the out-of-plane bending mode vibration of the C‒N ring in a basic structural heterocycles of g-C_3_N_4_ (Gao et al., 2018). O‒H stretching vibrations, which is caused by the hydroxyl groups on the surface and the water molecules that have been adsorbed, is responsible for the appearance of absorption peaks at 3401 cm^−1^ and 1628 cm^−1^. It is observed that the distinctive g- C_3_N_4_ absorption peaks in the FTIR spectra of the TNSs@Au/g- C_3_N_4_ increased, however the intensity of the absorption peaks below 1000 cm^−1^ is wider and dropping. The distinct absorption peaks of g-C_3_N_4_ were clearly increased in FTIR spectra of the TNSs@Au/g-C_3_N_4_ catalyst, while the strength of the absorption peaks under 1000 cm^−1^ became wider and feeble, ascribed to the Ti‒O‒Ti and Ti‒O‒C stretching vibrations (Cao et al., 2019). The chemical bond of Ti‒O‒C is built up *via* surface hydroxyl groups (Ti‒OH) of TNSs@Au and the remaining carboxylic groups (COOH) C‒N=C bonds of g-C_3_N_4_, which is consistent with the XPS studies. To evaluate the effectiveness of photocatalytic degradation of organic contaminants, the optical absorption range and efficiency over photocatalysts are essential. Figure 4B displays the findings related to the UV-vis spectrum absorption ranges of g-C_3_N_4_, TNSs, TNSs@Au, and TNSs@Au/g- C_3_N_4_ photocatalysts. The absorption range of TNSs appears to be longer than that of UV light alone because of an abundance of Ti-O-Ti bonds on (001) facet and the surface-unsaturated titanium atoms (Sun et al., 2012). Due to small band gap, pure g-C_3_N_4_ NSs appeared to extend into the visible region with an approximately 457 nm absorption edge. TNSs@Au photocatalyst had a discernible absorption band around 570 nm as an effect of the surface plasmon resonance (SPR) effect of Au NPs (Bian et al., 2014). To boost the photocatalytic processes, the supported Au NCs’ SPR effect promotes light absorption and photoinduced carrier separation. The absorption intensity of TNSs@Au/g-C_3_N_4_ in visible light was enhanced through addition of g-C_3_N_4_ NSs compared to TNSs@Au catalyst, and its absorption edge was slightly shifted towards higher wavelength. That serves as a clear demonstration of how g-C_3_N_4_ NSs improve the effectiveness of visible light absorption. Figure 4C presented the outcomes of applying the Kubelka-Munk function to further analyze the bandgap of photocatalysts (López and Gómez, 2012). The spectral response of the photocatalyst was used to calculate the parameter absorption coefficient (*a*), Planck constant (h), light frequency (ν), a constant (A), and band gap energy (E_g_) of (αhν) ^n^ = A (hν-Eg) equation. TNSs showed smaller E_g_ value of 2.94 eV than the commercial TiO_2_ (3.2 eV). The E_g_ value of g-C_3_N_4_ NSs was only 2.4 eV due to the characteristics of g-C_3_N_4_ NSs, which have a relatively narrow band gap. TNSs@Au/g-C_3_N_4_ photocatalysts having improved visible-light absorption efficiency were therefore expected to increase the rate at which organic contaminants were degraded. To relate the electric current density under situations of light and darkness, some on/off visible light irradiation cycles were used to measure the transient photocurrent responses of TNSs, g- C_3_N_4_, TNSs@Au, and TNSs@Au/g- C_3_N_4_ photocatalysts (see Figure 4D). The photocurrent ascends as soon as the light irradiation was turned on, and it quickly fell to zero once the light radiation was switched off. The photoelectric current responses of the catalysts clearly improved with the addition of supported Au NCs, demonstrating that they can boost surface photocurrent density. The surface photocurrent density of TNSs@Au/g-C_3_N_4_ catalysts continues to rise after adding additional wrapping g- C_3_N_4_ nanolayers, demonstrating the exceptional charge separation capabilities in our proposed system. The observations indicate that supported Au NPs own a high degree of surface enhancement in photoexcited e-h pair, which suggests that the creation of a composite TNSs@Au/g-C_3_N_4_ system may effectively control the recombination and lift the separation efficiency of photoinduced electrons and holes.

**FIGURE 5:**
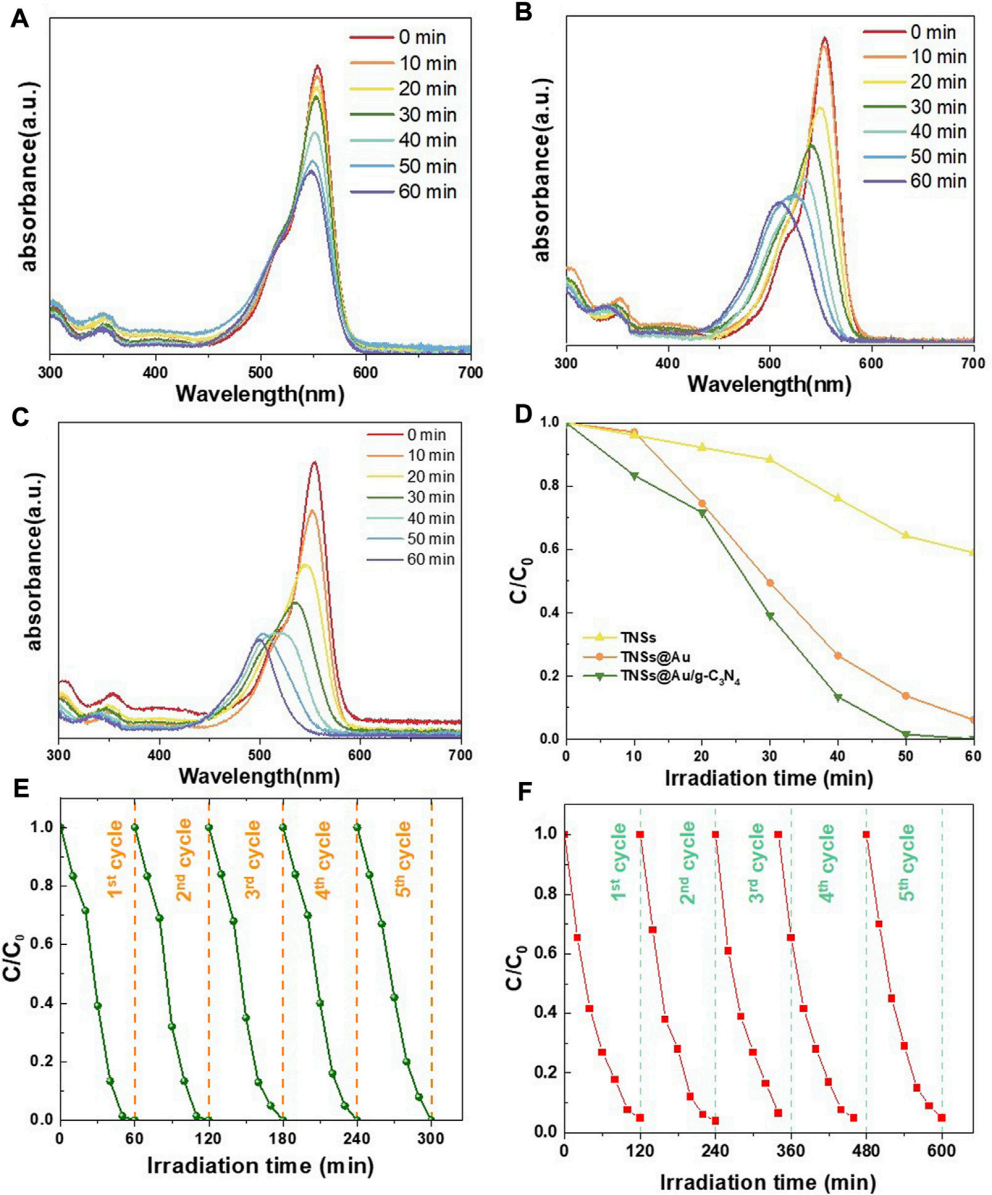
RhB aqueous solution's absorption spectrum degradation when exposed to **(A)** TNSs, **(B)** TNSs@Au, and **(C)** TNSs@Au/g-C_3_N_4_ based on the visible light irradiation time; **(D)** RhB photocatalytic degradation comparison under prepared catalysts; **(E and F)** Recycled photocatalytic degradation of RhB and MO.

As represented in Figure 5, the photocatalytic activity of the generated catalysts was assessed using photocatalytic decolorization of aqueous RhB solution under visible-light irradiation (420 nm) at room temperature. The spectral variations of RhB in the TNSs, TNSs@Au, and TNSs@Au/g-C_3_N_4_ photocatalysts in visible light degradation are depicted in Figures 5A–C respectively. The decline in absorbance at 554 nm, which emphasizes the degradation of RhB on the generated photocatalyst, indicates the photocatalytic activeness. With respect to the TNSs (Figure 5A), the TNSs@Au (Figure 5B) showed a significant increase in the RhB degradation due the Au content on the (001) active edge-sites that improved the efficiency of RhB photodegradation. Figure 5C illustrated the significant increase in the reduction in absorbance at 554 nm for the TNSs@Au/g-C_3_N_4_ photocatalyst. The ratio of residual RhB (C/C_0_) was used to assess the degradation performance, where C_0_ is the dye concentration in the dark after adsorption, and C is the dye concentration after radiance for time t. Comparative analysis of photocatalytic degradation processes of TNSs, TNSs@Au, and TNSs@Au/g-C_3_N_4_ photocatalysts is shown in Figure 5D. It is evident from the results that when the TNSs were introduced, a slight increase in the RhB degradation was detected. Due to the fact that the conduction band (CB) of TNSs is smaller than the RhB oxidation potential, excited RhB molecules transfer electrons to TNSs while also deteriorate subsequently. However, the TNSs@Au showed a significant increase than bare TNSs when we introduced it to the RhB aqueous solution. With the aid of TNSs@Au photocatalysts, the SPR effect of Au has improved the optical absorption in the visible photo-range and accounts for the improved photocatalytic activity of TNSs@Au. Due to the high degree of crystallinity of Au NCs, which prevents excited electrons and holes from recombining and so improves the photocatalytic performance, the barrier to electron migration is quite low. It was fascinating to see that 99.4% of the RhB in TNSs@Au/g-C_3_N_4_ structure decayed in 60 min under visible light illumination, considerably outpacing the TNSs’ capabilities. These results demonstrate that the g-C_3_N_4_ 2D geometry and integration of Au NCs active sites considerably improved the photocatalytic activity.

Methyl orange (MO) was also chosen as the degradation subject to test the photocatalytic activity in order to evaluate adaptability and effectiveness of produced photocatalyst. As anticipated, the MO degradation results followed the trend of RhB (Supplementary Figure S3). The best degradation effect was achieved by TNSs@Au/g-C_3_N_4_, and the corresponding MO degradation rate was 95.1 percent in 120 min. The observed TNSs and TNSs@Au degradation rates were 7.2 percent and 79.5 percent, respectively. It was worth to see that the photodegradation efficiencies of the TNSs@Au and TNSs@Au/g-C_3_N_4_ catalysts were 11 and 13.2 times greater than bare TNSs, noting that TNSs catalyst had almost negligible effect on the aqueous MO solution under visible-light. The hetero-interfacial photocatalyst is constructed with a synergistic interaction among TiO_2_, Au, and g-C_3_N_4_ wich produced a high separation efficiency of photoinduced electron-hole pairs and highest degradation rate. These findings showed that modifying TNSs using Au NCs and coupling to g-C_3_N_4_ semiconductor is a viable tactic that can considerably increase the harvesting of visible light irradiation to degrade organic contaminants. This is widely acknowledged that the LSPR of Au might increase the photocatalytic effectiveness by producing “hot electrons” that participated in chemical reactions in addition to broadening the visible light absorption (Zeng et al., 2020; Lv et al., 2022). Two electron-flow channels are present in TNSs@Au under simulated solar light. One is from TiO_2_ to Au NCs because of the potential difference among TiO_2_ and Au. The other is caused by the injection of “hot electrons” from Au NCs to TiO_2_. These two approaches conflict with one another, reducing the effectiveness of charge separation and improving photocatalytic performance of TNSs@Au only somewhat above TNSs. When this structure is interfacial-engineered by g-C_3_N_4_, the output photoactivity of TNSs@Au/g-C_3_N_4_ is credited to the coupled junctions in TNSs@Au/g-C_3_N_4_, that has exciting separation efficiency for photoinduced electron-hole pairs in photocatalyst, as well as to the injection of “hot electrons” brought on by the LSPR effect of Au, that added surplus electrons to the reaction (Sun et al., 2015; Cheng et al., 2022). At a particular wavelength of irradiation, the surface electrons of Au NCs are stimulated and start to oscillate collectively with the incident electromagnetic field, producing “hot electrons” in the surface plasma (SP) state. The CBs of the adjacent TiO_2_ and g-C_3_N_4_ received these “hot electrons,” which were subsequently transmitted to them, respectively. Interestingly, no remarkable decrease in the photocatalytic performance was observed after five successive recycles of photodegradation of RhB (Figure 5E) and MO (Figure 5F), confirming that TNSs@Au/g-C_3_N_4_ nanostructures are photostable during the photocatalytic degradation of the organic dye molecules.

**FIGURE 6:**
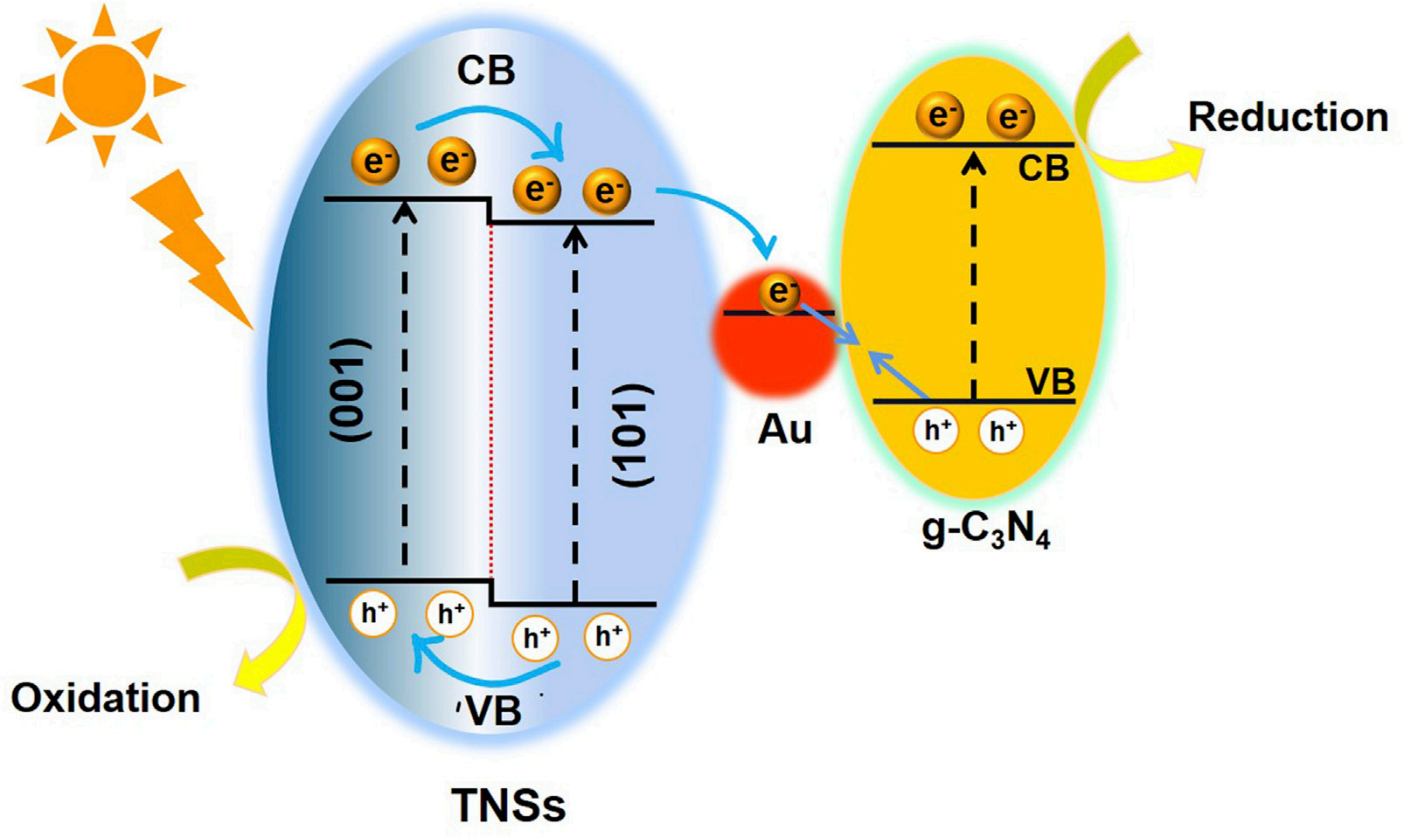
Schematic illustration of energy-band diagram and electron–hole transfer in the TNSs@Au/g-C_3_N_4_ photocatalyst.

Due to the strong interaction between the Au NCs and TNSs, as well as the lower Fermi level of Au in relation to TiO_2_, the g-C_3_N_4_ preferred to interact with the Au NCs. The interface-interface interaction between Au-g-C_3_N_4_ was later restricted because of the nanosized Au. In the meantime, (101) facets of TNSs, which are other electron-rich regions, saw an extension of the interfacial contact caused by g-C_3_N_4_. Due to their intimate junction and the extended interfacial contact, TiO_2_ (101) facets and g-C_3_N_4_ will produce a direct Z-scheme charge transfer in addition to the Z-scheme described above. The TiO_2_ → Au → g-C_3_N_4_ Z-scheme design controls the charge transfer process, nevertheless, because of exciting electron trapping capabilities of Au NCs. Further growth of g-C_3_N_4_ could only take place on the (001) facets once the surface of (101) facets had been fully covered. We hypothesized a photocatalytic mechanism (see Figure 6) using the charge-transfer measurements as a basis and degradation studies. Under UV light irradiation, the electrons on the VBs of TiO_2_ and g-C_3_N_4_ were both energized and transported to their respective CBs. Due to the close heterointerface formed between the Au NCs and TNSs and the lower Fermi level of Au than that of TiO_2_, the electrons from the CB of TiO_2_ flowed into Au and later recombined with the holes from the VB of g-C_3_N_4_. This particular structure conserved the highly oxidizing holes in the VB of TNSs and highly reducing electrons in the CB of g-C_3_N_4_. A Z-scheme charge transfer mechanism was created as a result. We detailed the recent research on 2D-based Z-scheme photocatalysts in the degradation of RhB and MO to further highlight the advantages of our photocatalytic system (Supplementary Table S1, Supporting Information). Our proposed design performed noticeably better than existing 2D Z-scheme photocatalysts in the degradation of organic pollutants. This demonstrates the system's tremendous ability to photodegrade organic contaminants.

## Conclusion

In conclusion, we have designed and fabricated an effective TNSs@Au/g-C_3_N_4_ ternary nanocomposite heterojunction through a controlled hydrothermal synthesis strategy and programmable calcination to facet-selective growth of edge sites deposited Au on 2D TNSs followed by encapsulation with g-C_3_N_4_. Au NCs, that can capture electrons from TNSs, create surplus electrons *via* the LSPR effect for effective photoreduction, and provides a strong affinity to make strong interfacial contact with g-C_3_N_4_ sheets. This engineered Z-scheme photocatalytic system is comprised of two photochemical schemes, precisely TNSs and g-C_3_N_4_ bridged with Au NCs as the electron transfer channel. This structure provided excellent active sites for redox reactions and prevented charge recombination in the structure while maintaining the vastly bare surface to the maximum possible level. Due to the SPR produced by Au NCs, the TNSs@Au/g-C_3_N_4_ nanocomposites significantly shifted light absorption to a longer wavelength. The exposed (001) and (101) surface/inter-facet junction and the Au/TNSs interfacial heterojunction enable the effective charge separation, coupled with 2D overlay of g-C_3_N_4_ sheets synergistically offering tremendous reactive sites for the potential photocatalytic dye degradation in the Z-scheme photocatalyst. Photoinduced electrons in TNSs are moved to Au NCs and recombined with the holes produced in g-C_3_N_4_, producing a Z-scheme charge transfer channel, according to photocurrent response measurements. This work will stimulate thinking to selectively design and construct other 2D/0D/2D Z-scheme photocatalytic systems with strong photocatalytic.

## Data Availability

The original contributions presented in the study are included in the article/Supplementary Material, further inquiries can be directed to the corresponding author.
