# Supplementary material for: Semiconductor-metal-semiconductor TiO2@Au/g-C3N4 interfacial heterojunction for high performance Z-scheme photocatalyst
Source: Front Chem. 2022 Oct 21;10:1050046. doi: 10.3389/fchem.2022.1050046 (PMC9646487; doi:10.3389/fchem.2022.1050046)
Supplement: Supplementary file 1 [file DataSheet1.docx]

Supplementary Material

Semiconductor-Metal-Semiconductor TiO_2_@Au/g-C_3_N_4_ interfacial Heterojunction for High Performance Z-Scheme Photocatalyst

Tingkai Hong^1†^, Shoaib Anwer ^2†^, Ju Wu^3^_,_ Chonghai Deng^4^_,_ Hongmei Qian^5*^

^1^ School Energy Materials and Chemical Engineering, Hefei University, Hefei 230601, China

^2^ Department of Mechanical Engineering, Khalifa University of Science and Technology, Abu Dhabi 127788, United Arab Emirates

^3^ Key Laboratory of Biomimetic Sensor and Detecting Technology of Anhui Province, West Anhui University, Luan 237012, China

^4^ School Energy Materials and Chemical Engineering, Hefei University, Hefei 230601, China

^5^ Department of Architecture and Civil Engineering, West Anhui University, Luan 237012, China

**^†^** These authors contributed equally to this work

*** Correspondence:**H. M. Qian
[hmqian0621@163.com](mailto:hmqian0621@163.com)


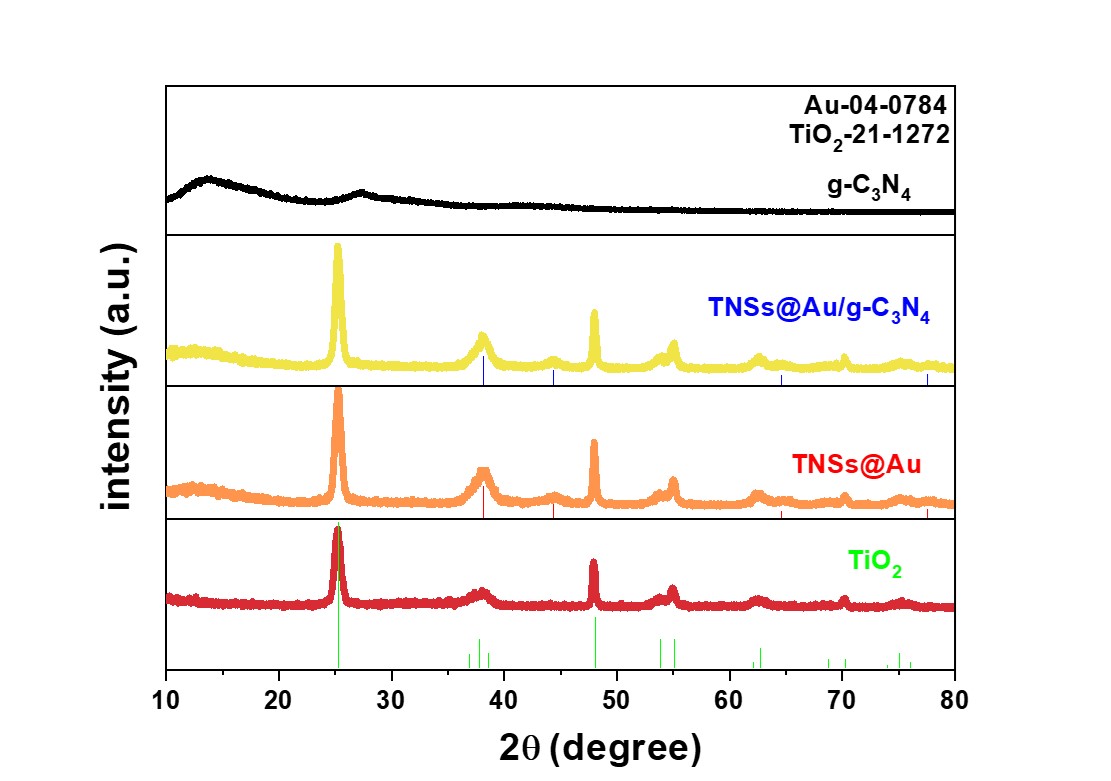


**Figure S1.** XRD patterns of the prepared samples.


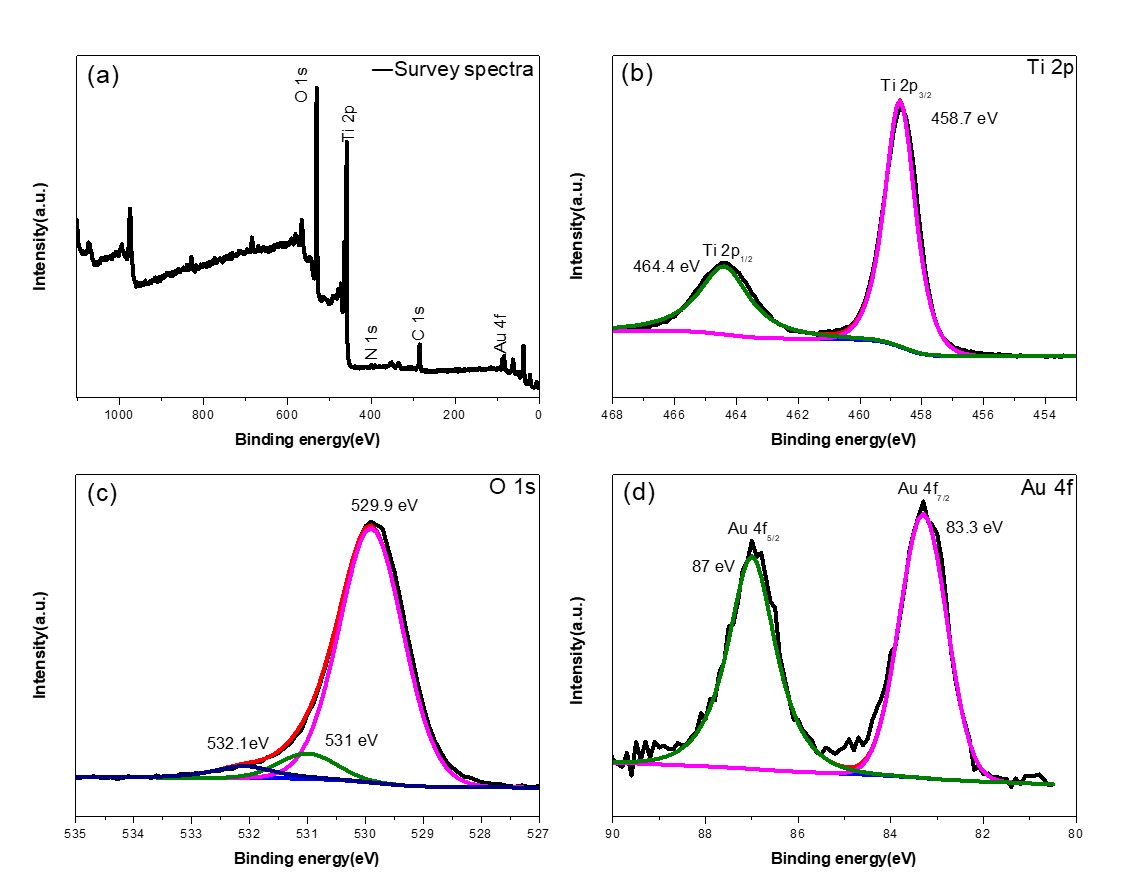


**Figure S2.** High-resolution XPS spectra of as-prepared TNSs@Au photocatalyst: (a) full scan survey, (b) Ti 2p, (c) O 1s, (d) Au 4f.


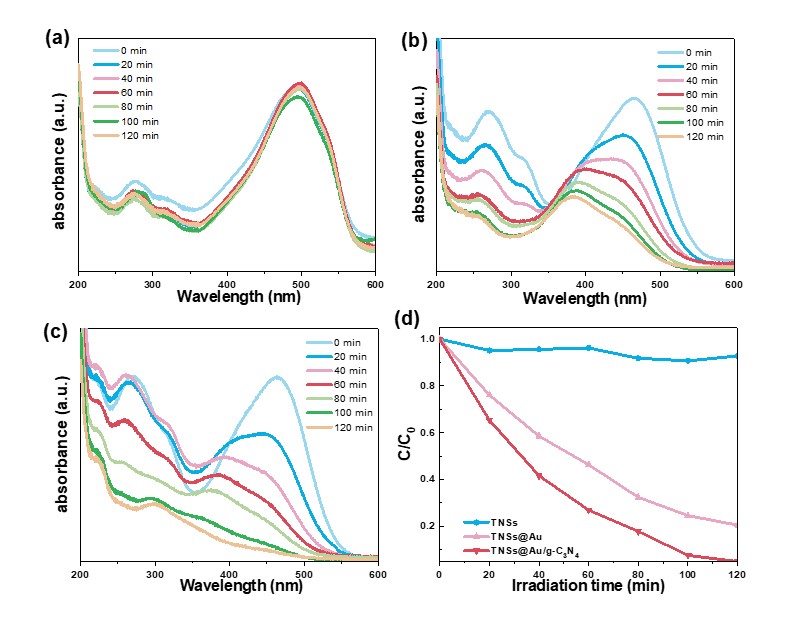


**Figure S3.** Absorption spectral degradation of MO aqueous solution in the presence of (a) TiO_2_, (b) TNSs@Au, and (c) TNSs@Au/g-C_3_N_4_ as a function of irradiation time (under visible light); (d) Comparison of MO photocatalytic degradation.


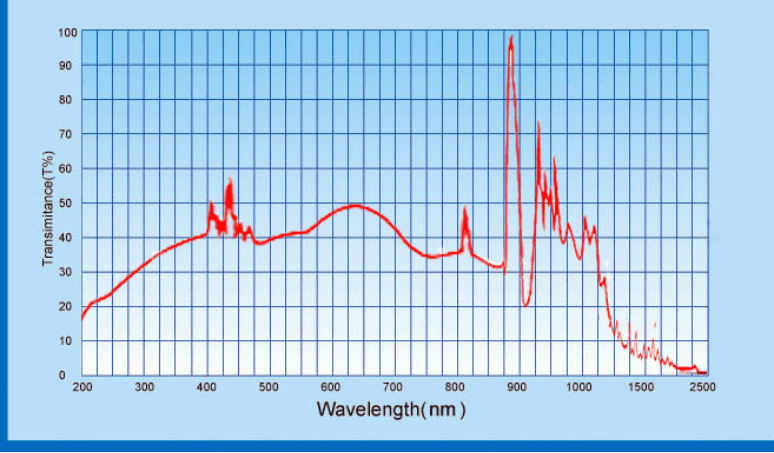


**Figure S4**. The spectrum obtained from the datasheet of Xenon light source (150 W) used.

**Table S1:**

Comparison of the as-obtained photocatalyst for the RhB & MO dye photo-degradation (%) with earlier reported photocatalysts.

| Catalysts | Light source | Dosage | Degradation target | Irradiation time (min) | Degradation (%) | Ref. |
| --- | --- | --- | --- | --- | --- | --- |
| g-C_3_N_4_(Ag)/Gr/TiO_2_ | Xenon (λ ≥ 400 nm) | 100 mg | RhB | 120 | 99.7 | [1] |
| g-C_3_N_4_/TiO_2_/CuO | 500 W Xenon | 50 mg | RhB | 120 | 90.3 | [2] |
| Ag-TiO_2_/PP | Xenon | 50 mg | MO | 180 | 81.4 | [3] |
| TiO_2_/g-C_3_N_4_ | 400 W (UV-cut off) | 50 mg | RhB | 90 | 93 | [4] |
| TiO_2_/g-C_3_N_4_ | 300 W Xenon | 50 mg | RhB | 60 | 95 | [5] |
| TiO_2_/g-C_3_N_4_ | 300 W Xenon | 50 mg | MO | 60 | 66 | [5] |
| TiO2@g-C3N4/Co3O4 | Xenon  (300 W) | 50 mg | MO | 60 | 97.8 | [6] |
| g-C_3_N_4_/Ag-TiO_2_ | 500 W Xenon (λ ≥ 420nm) | 50 mg | RhB | 105 | 92.7 | [7] |
| TiO_2_/Au/g-C_3_N_4_ | 280W Xenon lamp (λ ≥ 420nm) | 50 mg | RhB | 60 | 99.4 | This work |
| TiO_2_/Au/g-C_3_N_4_ | 280xenon lamp (λ ≥ 420nm) | 50 mg | MO | 120 | 95 | This work |

[1] S. Xue, H. Li, F. Cao, Y. Cao, X. Yue, Preparation of g-C_3_N_4_ (Ag)/Gr/TiO_2_ Z-scheme photocatalyst with enhanced reduction property and the efficient degradation of rhodamine B, Journal of Alloys and Compounds 898 (2022) 162759.

[2] R. Rajendran, S. Vignesh, S. Suganthi, V. Raj, G. Kavitha, B. Palanivel, M. Shkir, H. Algarni, g-C_3_N_4_/TiO_2_/CuO S-scheme heterostructure photocatalysts for enhancing organic pollutant degradation, Journal of Physics and Chemistry of Solids 161 (2022) 110391.

[3] A. Sabir, T.A. Sherazi, Q. Xu, Porous polymer supported Ag-TiO_2_ as green photocatalyst for degradation of methyl orange, Surfaces and Interfaces 26 (2021) 101318.

[4] S.R. AR, H.M. Wilson, B.M. Momin, U.S. Annapure, N. Jha Revision, TiO_2_ nanosheet/ultra-thin layer gC _3_N_4_ core-shell structure: bifunctional visible-light photocatalyst for H_2_ evolution and removal of organic pollutants from water, Applied Surface Science 528 (2020) 146930.

[5] S. Feng, F. Li, Photocatalytic dyes degradation on suspended and cement paste immobilized TiO_2_/g-C_3_N_4_ under simulated solar light, Journal of Environmental Chemical Engineering 9(4) (2021) 105488.

[6] B. Yu, F. Meng, M.W. Khan, R. Qin, X. Liu, Synthesis of hollow TiO_2_@ g-C_3_N_4_/Co_3_O_4_ core-shell microspheres for effective photooxidation degradation of tetracycline and MO, Ceramics International 46(9) (2020) 13133-13143.

[7] G. Sui, J. Li, L. Du, Y. Zhuang, Y. Zhang, Y. Zou, B. Li, Preparation and characterization of g-C_3_N_4_/Ag–TiO_2_ ternary hollowsphere nanoheterojunction catalyst with high visible light photocatalytic performance, Journal of Alloys and Compounds 823 (2020) 153851.
